# Development, Preparation, and Biomedical Applications of DNA-Based Hydrogels

**DOI:** 10.3389/fbioe.2021.661409

**Published:** 2021-06-02

**Authors:** Xueting Jian, Xiaoyi Feng, Yuning Luo, Fangjie Li, Junyan Tan, Yuli Yin, Yang Liu

**Affiliations:** Hunan Provincial Key Laboratory of Tumor Microenvironment Responsive Drug Research, Hunan Province Cooperative Innovation Center for Molecular Target New Drug Study, College of Pharmacy, University of South China, Hengyang, China

**Keywords:** DNA, pure DNA-based hydrogel, DNA-based hybrid hydrogel, crosslinking, biomedical application

## Abstract

Hydrogels have outstanding research and application prospects in the biomedical field. Among them, the design and preparation of biomedical hydrogels with deoxyribonucleic acid (DNA) as building blocks have attracted increasing research interest. DNA-based hydrogel not only has the skeleton function of hydrogel, but also retains its biological functions, including its excellent selection specificity, structural designability, precise molecular recognition ability, outstanding biocompatibility, and so on. It has shown important application prospects in the biomedical field, such as drug delivery, biosensing, and tissue engineering. In recent years, researchers have made full use of the characteristics of DNA molecules and constructed various pure DNA-based hydrogels with excellent properties through various crosslinking methods. Moreover, via introducing functional molecules or elements, or combining with other functional materials, a variety of multifunctional DNA-based hybrid hydrogels have also been constructed, which expand the breadth and depth of their applications. Here, we described the recent development trend in the area of DNA-based hydrogels and highlighted various preparation methods of DNA-based hydrogels. Representative biomedical applications are also exemplified to show the high performance of DNA-based hydrogels. Meanwhile, the existing problems and prospects are also summarized. This review provided references for the further development of DNA-based hydrogels.

## Introduction

Hydrogel is a three-dimensional (3D) polymer composite system composed of water and crosslinked polymers. It has similar physical and chemical properties to human tissues and has great application prospects in the biomedical field, such as drug delivery, tissue engineering, and biosensing (Ahmed, 2013; Chai et al., 2017; Daly et al., 2020; Spicer, 2020). In recent years, various hydrogels with special structures and functions have been designed and developed. Among them, the use of deoxyribonucleic acid (DNA) as building blocks to prepare hydrogels has attracted the attention of many researchers (Shao et al., 2017; Li F. et al., 2019; Chen et al., 2020). DNA, an essential macromolecule for the normal growth and development of most organisms, is the main carrier of coding, storage, and transmission of genetic information. DNA generally contains two deoxyribose nucleotide single chains, each of which is composed of monomers containing four bases, including adenine (A), guanine (G), cytosine (C), and thymine (T), so DNA can also be used as an anionic block copolymer in the biomedical field. According to the specific pairing principle of bases on the chain, two single chains can form a stable double-helix structure connected by hydrogen bonding. The highly selective recognition of these bases and the designability of sequence coding endow DNA with powerful assembly ability. Besides, the outstanding biocompatibility and biodegradability, long persistence length, and ease of modification of DNA aroused great interest of researchers in the biomedical field in DNA assembly materials (Jorge and Eritja, 2018; Seeman and Sleiman, 2018; Hu and Niemeyer, 2019). Recently, a variety of DNA assembly materials with precise structure and rich functions, especially DNA-based hydrogels, have been emerging, whose applications have gradually expanded from life science to the biomedical field (Li et al., 2016; Kahn et al., 2017; Zhou et al., 2020). Nagahara and Matsuda (1996) covalently introduced oligonucleotides into the side chain of polymer chains and prepared an intermolecular crosslinked hydrogel through hydrogen bonding of bases. This was the first report on DNA-based hybrid hydrogels to be designed and synthesized. In Um et al. (2006) used branched DNA as building blocks to form pure DNA-based hydrogels for the first time under physiological conditions through ligase mediation, which opened up a new direction for the research of DNA-based hydrogels. Later, they used linear long-chain DNA as building block and constructed a novel pure DNA-based hydrogel through physical entanglement for the first time (Lee et al., 2012). Through decades of research and development, DNA-based hydrogels have become a hotspot in the biomedical field now.

**FIGURE 1:**
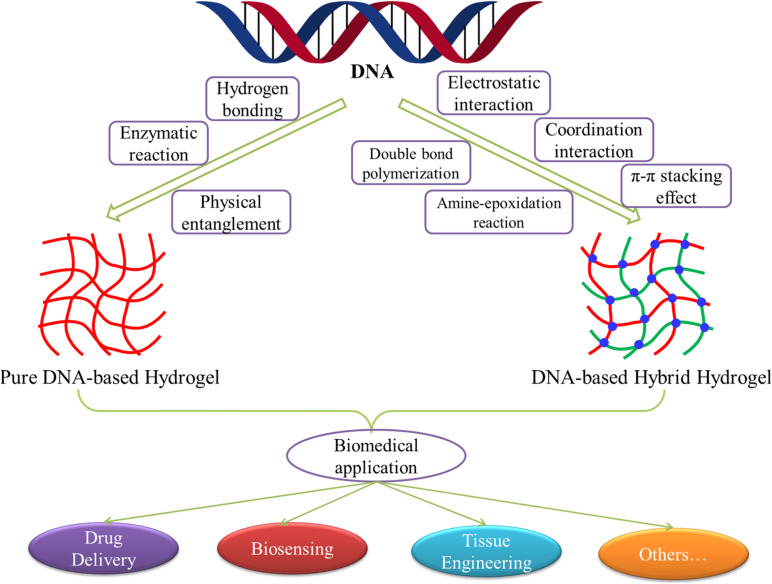
Schematic diagram of the development, preparation, and biomedical applications of DNA-based hydrogels.

A host of studies has shown that DNA-based hydrogels not only retain the biological functions of DNA but also have the skeleton functions of hydrogels. Besides the general characteristics of hydrogels, it also has the advantages of good selection specificity, structural designability, and precise molecular recognition ability. Therefore, DNA-based hydrogels have shown excellent development and clinical application prospects in the fields of drug delivery, biosensing, tissue engineering, protein engineering, and cell imaging (Gačanin et al., 2020; Li et al., 2020; Morya et al., 2020). With the continuous deepening of the research, by using the characteristics of DNA molecules, introducing other functional molecules or elements into DNA, or combining with other functional materials, researchers have developed various crosslinking methods for the preparation of pure DNA-based hydrogels or DNA-based hybrid hydrogels. Moreover, the application extent and scope of DNA-based hydrogels are also expanding, and the research depth is gradually changing from basic research to solving practical problems in the biomedical field. In this review, we summarized and elaborated the development, preparation, and biomedical applications of pure DNA-based hydrogels and DNA-based hybrid hydrogels in recent years (Figure 1). By analyzing and prospecting the existing problems and future development trends of the DNA-based hydrogels, we hoped to provide references for the subsequent research of DNA-based hydrogels.

## The Preparation of Pure DNA-Based Hydrogels

Pure DNA-based hydrogels are a type of hydrogels composed entirely of deoxyribonucleotide chains. They are usually formed through hydrogen bonding, physical entanglement, or enzymatic reaction between chains (Figure 1). Because of the precise structural controllability, specific responsiveness, and excellent biodegradability, pure DNA-based hydrogels play a considerable role in the construction of sensitive smart materials for drug delivery, biosensing, and other biomedical applications.

### Hydrogen Bonding

The DNA contains A, G, C, and T, four bases that have high specificity of complementary pairing. Consequently, the crosslinking of complementary base sequences on adjacent DNA by hydrogen bonding has become the main forming way of pure DNA-based hydrogels. The first DNA-based hydrogel reported in the world was exactly crosslinked by hydrogen bonding between bases using DNA as crosslinking agents (Um et al., 2006). These kinds of hydrogels usually have a stable and controllable structure, which demonstrates numerous extraordinary behaviors, such as self-healing and dynamic response, and are widely used in practice.

**FIGURE 2:**
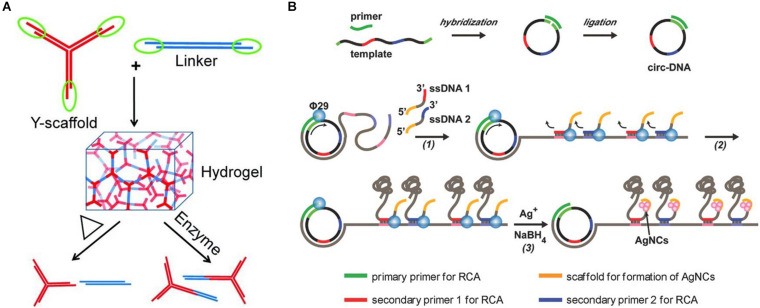
**(A)** Schematic diagram of the preparation of pure DNA-based hydrogel by hydrogen bonding of Y-scaffold and linker. Reproduced with permission from Xing et al. (2011). Copyright (2011) Wiley-VCH. **(B)** Schematic diagram of the preparation of biofunctional DNA-based hydrogel by physical entanglement. Reproduced with permission from Geng et al. (2018). Copyright (2018) Wiley-VCH.

Representative research designed a kind of Y-scaffold and linker to prepare pure DNA-based hydrogel based on DNA self-assembly (Xing et al., 2011). The Y-scaffold is a Y-shaped DNA nanostructure assembled from three kinds of single-stranded DNA (ssDNA) strands, whereby each strand has a sticky end segment to hybridize with its complementary part on the linker and two segments to hybridize with the two other strands (Figure 2A). The linker is a linear duplex formed by two ssDNAs and contains two sticky ends. The sticky ends of the Y-scaffold and linker can be complementary to each other by hydrogen bonding, and this hybridization will lead to the formation of the hydrogel. This pure DNA-based hydrogel could reversibly respond to the thermal stimulus by switching between the gel and sol state across a transition temperature, as well as respond to enzymes when restriction sites are inserted into the building blocks, which provide a new type of smart materials for diverse biomedical applications.

Another research also designed a universal modular strategy for the construction of pH-activated pure DNA-based hydrogels from Y-shaped DNA nanostructures (Lu et al., 2018). Through ingenious design, they synthesized six kinds of Y-shaped DNA modules that could be complementary to each other and form two kinds of triplex structures based on protonated cytosine–guanine–cytosine (C–G⋅C^+^) and thymine–adenine–thymine (T–A⋅T). As the C–G⋅C^+^ bridges could form at pH 5.0 and disassemble at pH 7.0, and the T–A⋅T bridges form at pH 7.0 and disassemble at pH 10.0, the DNA modules could be manipulated into three types of pH-activated pure hydrogels through the changes in pH value. These types of reverse hydrogels can respond to the acidity or alkalinity in different environments of the biosomes, which are particularly attractive in the biomedical field.

### Physical Entanglement

Because the single-stranded deoxyribonucleotide has bright flexibility, and the DNA molecules easily form specific spatial stereoscopic structures, the long-chain DNA molecules could easily have physical entanglement and form macroscopic hydrogels. This kind of pure DNA-based hydrogels usually exhibits an extraordinary mechanical performance and is reversible under the stimulation of the external environment, which is suitable for drug delivery and biosensing.

As mentioned previously, the first pure DNA-based hydrogel formed from linear long-chain DNA was precisely obtained through physical entanglement (Lee et al., 2012). The researchers used a special bacterial phage polymerase φ29 to elongate DNA strands by using ssDNA as a primer. According to the rolling cycle amplification (RCA) and the following multiple primer chain amplification, an enzymatic chain reaction takes place, and specific DNA long chains were formed. Then, a deformable pure DNA-based hydrogel metamaterial with a hierarchical structure could be formed through physical entanglement. Under the stimulation of H_2_O molecules, the DNA-based hydrogel could undergo free, fast, and reversible changes between liquid and solid states, which is expected to be applied in drug-controlled release, cell therapy, and other real-world application areas. Similarly, Huang et al. also used linear long-chain DNA and prepared a timesaving, labor-saving, and high-stability pure DNA-based hydrogel with high catalytic activity by physical entanglement (Huang et al., 2017). They first used guanine (G)–rich DNA templates to form repetitive long ssDNA chains by RCA amplification, which also would form DNA-based hydrogel by physical entanglement. Afterward, hemoglobin was loaded into this hydrogel structure, which made the DNA-based hydrogels have stable horseradish peroxidase (HRP)–like catalytic functions. By combining with glucose oxidase (GOx), this DNA-based hydrogel can also be applied to the sensitive and portable detection of glucose.

Meanwhile, another representative research also used circular DNA as the template for RCA amplification (Geng et al., 2018). Differently, two new pieces of ssDNA that contained sticky ends of the cytosine (C)–rich domains were introduced into the RCA reaction as the secondary primers at the second round of RCA, resulting in the generation of massive DNA chains, which would weave into a 3D hydrogel network by entanglement (Figure 2B). Furthermore, because of the presence of the high affinity of Ag^+^ ions with cytosine, a type of silver nanocluster–doped DNA-based hydrogel that exhibits excellent fluorescent and strong antibacterial functions could also be formed. Similarly, Mao et al. also prepared a pure DNA-based hydrogel on the ITO (indium tin oxide) electrode through physical entanglement. After further incubation with hemin and toluidine blue O, the resulting hydrogel could be used as an electron transporter, which provided a potential application prospect in high-performance biosensors (Mao et al., 2020).

### Enzymatic Reaction

The traditional hydrogen bonding–based strategy for constructing DNA-based hydrogels usually requires long-enough sticky ends to stabilize hydrogels, which is inconvenient in practical applications. Some studies have shown that ligase could also connect DNA fragments with complementary sticky ends and even repair defects in double-stranded DNA (dsDNA) with 3′-hydroxyl and 5′-phosphate ends. Hence, the formation of DNA-based hydrogels by covalently crosslinking of ligases has attracted great interest from researchers.

The Luo group described previously first used branched DNA as building blocks to form pure DNA-based hydrogel through the efficient mediation of ligase under physiological conditions (Um et al., 2006). These hydrogels were obtained from three shapes of DNA structures: the X-shaped DNA, Y-shaped DNA, and T-shaped DNA, each arm of which contained complementary sticky ends with palindromic sequences. Under the action of T4 ligase, three DNA structures could be chemically crosslinked to form the macroscopic hydrogel. Drugs, proteins, and even live mammalian cells can be encapsulated *in situ* in this type of hydrogel. Through adjusting the initial concentration and type of DNA structures, the formed hydrogels could be finely tuned for specific applications. They further used polydimethylsiloxane as a module and formed a type of DNA-based hydrogel system (P-gel) for protein generation for the first time through the connection of X-DNA and linear plasmids catalyzed by T4 ligase (Park et al., 2009).

Bae et al. (2013) also utilized X-shaped DNA blocks to form pure DNA-based hydrogels under the action of T4 ligases. Differently, they used a new superhydrophobic layer prepared from zinc oxide–nanopatterned and heptafluorodethanethiol chemically treated gold surfaces for the preparation of hydrogels. Thus, microscale and spherical DNA-based hydrogels will be gained, which might show unique application prospects, such as pseudonucleus mimics. A similar method can also be used to prepare nanoscale DNA-based hydrogels for siRNA generation and interfere with protein expression (Song et al., 2018). The researchers first used the restriction enzyme *Bam*HI to linearize the plasmid DNAs, which carry the gene transcribing siRNA against the target mRNA. Then, the linearized plasmid DNAs were crosslinked with the X-shaped DNAs and formed an RNA-producing hydrogel under the influence of the T4 ligases. The results showed that this DNA-based gel has superior stability, cellular uptake efficiency, and silencing effect, which provides an ideal platform technology for an efficient RNA interference system.

## The Preparation of DNA-Based Hybrid Hydrogels

With the development of research, various novel functional materials or elements have been widely introduced into DNA-based hydrogels, which greatly enriches and expands the function and application of DNA-based hydrogels. According to different crosslinking and forming methods, these DNA-based hybrid hydrogels can be divided into two categories: physical crosslinked hydrogels and chemical crosslinked hydrogels. Physical crosslinked DNA-based hybrid hydrogels are mainly formed by physical interactions between DNA building blocks, such as electrostatic interaction, coordination interaction, π–π stacking effect, and so on (Figure 1). These crosslinking reactions are relatively simple and fast, and the formed DNA-based hydrogel often shows excellent responsiveness. While chemical crosslinked DNA-based hydrogels, because of their internal covalently bonded connection, have relatively stable structure and strong strength, thereby expanding the application scope of DNA-based hybrid hydrogels in the biomedical field. Common chemical bonds or chemical reactions involved double-bond polymerization, amine–epoxidation reaction, and so forth.

### Electrostatic Interaction

Because the bases of the DNA chain have many phosphate groups, DNA generally shows electronegativity. Thus, it is very easy to crosslink DNA with the positively charged molecules through strong electrostatic interactions to form hybrid hydrogels. The first electrostatically crosslinked DNA-based hydrogel was prepared through mixing ssDNA or dsDNA with cationic CTAB or lysozyme by interfacial diffusion, which does not need additional crosslinking agent and solvent (Morán et al., 2007).

**FIGURE 3:**
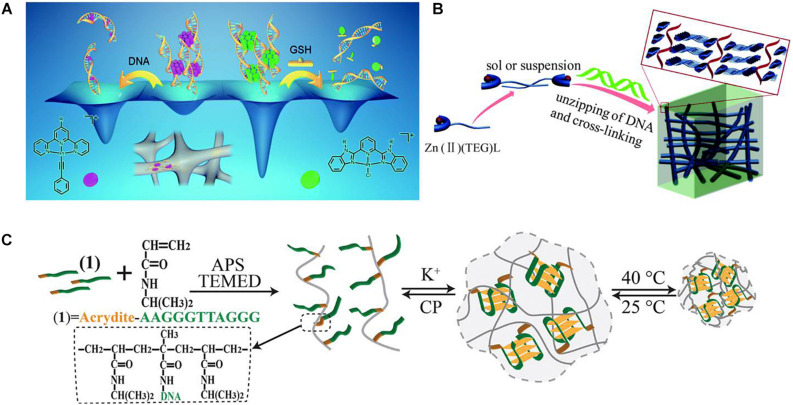
**(A)** Schematic diagram of using platinum(II) complexes as electrostatic crosslinking agents for the construction of DNA-based supramolecular hydrogels. Reproduced with permission from Zhang et al. (2020). Copyright (2020) The Royal Society of Chemistry. **(B)** Schematic diagram of using Zn^2+^ complexes for the preparation of DNA-based instant hydrogels by coordination interaction. Reproduced with permission from Geng et al. (2019). Copyright (2019) The Royal Society of Chemistry. **(C)** Schematic diagram of the preparation of K^+^-stabilized G-quadruplex–crosslinked DNA-based dual-stimuli-responsive PNIPAM hydrogel. Reproduced with permission from Lu et al. (2015). Copyright (2015) The Royal Society of Chemistry.

While Zhang and Yam used platinum(II) complex as an electrostatic crosslinking agent for the construction of DNA-based supramolecular hydrogel (Zhang and Yam, 2020). As shown in Figure 3A, after simply mixing of some unique platinum(II) complexes, such as alkynylplatinum(II) terpyridine, with dsDNA in an aqueous solution, the platinum(II) complexes may first stack into columnar phases by metal–metal and π–π stacking, and then the formed columnar phases with positive charges could crosslink with the negatively charged DNA strands to form supramolecular hydrogels with fibrous network structures, which shows good luminescence properties and excellent processability. These DNA-based hydrogels also show tunable gel-to-sol transition behaviors. Because of the presence of excessive DNA in the hydrogels, platinum(II) will intercalation into DNA base pairs subsequently, which would break the stacking phases and cause the hydrogel to dissociate. Also, if chloro(2,6- bis(benzimidazol-20-yl)pyridine)platinum(II) ([Pt(bzimpy)Cl]^+^) complexes are used as crosslinking agents, the second pathway of intercalation of platinum(II) into base pairs will be blocked; thus, the formed hydrogels could be able to stay steady. But this type of DNA-based hydrogels could also dissociate reversibly by the ligand exchange reaction of the chloro ligand in [Pt(bzimpy)Cl]^+^ with glutathione.

Nanoparticles could also be used to construct DNA-based hybrid hydrogels by electrostatic interaction, which endows the formed hydrogels with good mechanical properties, stability, and nanosize effect. Yang et al. (2013) utilized electrostatic interactions between clay nanometer crystals and dsDNA with higher charge densities and fabricated DNA-clay hybrid hydrogels for simulating biological cell microenvironment and studying life processes of DNA transcription and translation. The results showed that DNA transcription and translation activities had been strengthened and improved by this clay hydrogel (Yang et al., 2013).

### Coordination Interaction

Coordination compounds formed by the coordination interaction of atoms or ions with ligand molecules are widely used in life science, industrial production, and other fields. In the field of hydrogel, the preparation of polymer–metal hybrid hydrogels by coordination crosslinking of metal ions has also attracted wide attention. Many metal ions are easily prone to coordinate with the bases or phosphate groups on the DNA chain, thus crosslinking the adjacent DNA chains to form hybrid hydrogels (Clever and Shionoya, 2010). This hydrogel is particularly suitable for biomedical fields such as ion sensing detection, sensitive drug-controlled release, and so on.

Ag^+^ ions have a specific binding ability with cytosine (C) and could form C-Ag^+^-C complexes, so Ag^+^-crosslinked DNA hydrogels have drawn much attention. A representative study used Y-shaped DNA subunits with rich cytosine as building blocks and formed a type of Ag^+^-crosslinked reversible DNA-based hydrogel under the synergistic effect of complementary base pairs (Guo et al., 2014). Because cysteamine could form a more stable Ag^+^–cysteamine complex with Ag^+^ than cytosine, the Ag^+^ could be dissociated from the hydrogel after adding cysteamine, resulting in a reversible transition of this hydrogel from gel to sol state. This type of hydrogel could be applied in drug loading and controlled release. A similar method was also used to obtain a type of pH- and light-sensitive hydrogel assembled from 5-adenosine monophosphate (AMP) and Ag^+^ (Hu et al., 2017). They found that it is more efficient for the formation of hydrogels by increasing the concentration of Ag^+^ or decreasing the pH value. The results show that coordination of Ag^+^ with N1 and N7 of bases collaboration with π–π stacking of bases is the main driving force for the formation of this kind of hydrogel. These hydrogels could be used as excellent carriers for the encapsulation and enrichment of biomolecules.

Geng et al. designed and synthesized a series of Zn^2+^ complexes Zn(II)(TEG)L, which contain TEG tripyridine fluorescent ligands, sugar groups, and different ligand L anions (Geng et al., 2019). The existence of sugar groups could provide intermolecular hydrogen bonding and facilitate the self-assembly of Zn(II)(TEG)L complexes. The addition of dsDNA could increase the interaction between self-assembled Zn(II)(TEG)L complexes to form hydrogels because of the strong coordination between DNA and Zn^2+^ (Figure 3B). The gelation process could be achieved directly and rapidly at room temperature and can be controlled by controlling the anion ligand L. This research developed a simple and universal method for the rapid formation of metal composite DNA-based hydrogels and is highly promising in the field of biology and materials science.

Furthermore, the coordination interaction of metal ions with DNA could also be introduced into sensitive hydrogels. For example, Lu et al. (2015) designed a K^+^-stabilized G-quadruplex–crosslinked DNA-based dual-stimuli–responsive poly(*N*-isopropylacrylamide) (PNIPAM) hydrogel. As shown in Figure 3C, the acrydite-modified nucleic acid with the half subunit of the G-quadruplex was first used to polymerize with thermosensitive NIPAM monomers to yield nucleic acid–functionalized PNIPAM chains. After the introduction of K^+^ ions, because K^+^ could bridge the nucleic acid chains to form G-quadruplex structures, crosslinked PNIPAM hydrogel from PNIPAM chains could be prepared. If there is treatment of the hydrogel with kryptofix [2.2.2], the K^+^ ions would eliminate from the G-quadruplex bridges, resulting in the reverse dissociation of the hydrogel. Besides, because of the thermosensitivity of PNIPAM, the crosslinking density of the G-quadruplex–crosslinked PNIPAM hydrogel will increase with the thermal stimulus and induces the hydrogel-to-solid transition. By the incorporation of hemin, this hydrogel could also be a catalytic matrix for oxidation of aniline to form conductive polyaniline.

### π–π Stack Effect

π–π stacking is a special spatial arrangement of aromatic substances, and the resulting π–π stack effect is a weak interaction similar to hydrogen bonding, which is widespread in DNA double-helix structure. Thus, DNA-based hybrid hydrogels can also be crosslinked by the π–π stack interaction between DNA and aromatic compounds, such as graphene oxide (GO) derivatives. For instance, Xu et al. (2010) constructed a 3D hybrid hydrogel of GO sheets and ssDNA via π–π stacking. They simply mixed GO sheets with dsDNA aqueous solution at room temperature and allowed dsDNA *in situ* helices to form ssDNA by heating to 90°C. The obtained ssDNAs were able to lay on the surface of GO sheets via π–π stacking, thus making adjacent GO *in situ* connect to form stable DNA-based hydrogels. These hydrogels have interconnected microporous structures, showing good mechanical strength, environmental stability, dye loading rate, and self-healing property.

Because of the weak π–π stacking interaction between dsDNA and graphene, it is difficult to form stable hydrogels from dsDNA by general methods. Therefore, a simple self-assembly method for preparing GO/dsDNA composite hydrogels was developed (Kurapati et al., 2016). Unlike previous studies, the authors mixed GO and dsDNA of low concentrations directly in a special buffer solution containing sodium ions and formed a 3D hydrogel without heating. The authors believed the reason was that the cations in the buffer could bind to the phosphate ions in the dsDNA, as well as the carboxylate ions in the GO, thus reducing the electrostatic repulsion between dsDNA and GO. Therefore, stable hydrogels can be obtained under the π–π stacking between graphene and dsDNA.

### Double-Bond Polymerization

The chemical crosslinking reaction of DNA-based hybrid hydrogels formed by double-bond polymerization generally did not take place directly on the DNA blocks. Instead, the DNA blocks are usually covalently linked to polymerizable monomers first, and then the DNA-based hydrogels are obtained by the polymerization of these monomers (Khimji et al., 2013). Representative research prepared a type of DNA-based portable optical hydrogels oligonucleotide biosensors from acrylamide functionalized morpholino oligonucleotides (MO), acrylamide, and *N,N’-*methylene bis(acrylamide) (bis-acrylamide) by double-bond polymerization (Langford et al., 2019). MO consisted of two strands; the “sensor” strand was used for microRNA sequence detection, whereas the “blocker” strand was partially complementary to the sensor strand. Thus, MO could also be a physical crosslinking reagent, which would be selectively cleaved by a short target analyte based on microRNA and result in a distinct swelling response measured optically. These novel hydrogels can be further optimized to increase sensitivity of the biosensor systems. Similarly, to improve the signaling kinetics of hydrogels, a type of aptamer-functionalized hydrogel microparticles with diameters ranging from 10 to 50 μm was synthesized by emulsion double-bond polymerization technique from acrydite modified DNA, acrylamide, and bis-acrylamide (Helwa et al., 2012). Because this particular DNA aptamer contains seven hypothetical Hg^2+^ binding sites, Hg^2+^ would cause the aptamers to form a hairpin and give a fast change of fluorescence signal. Thus, the hydrogel microparticles can be used for fast visual detection of Hg^2+^. The hydrogel microparticles could also be spotted to form a microarray, which could be used for the detection of adenosine.

Moreover, a family of multicomponent DNA polymerization motor hydrogels with different polymer backbones by double-bond polymerization was recently reported (Shi et al., 2020). These motor hydrogels were prepared by crosslinking acrydite modified DNA duplex crosslinker with acrylamide, bis-acrylamide, poly(ethylene glycol) diacrylate, or gelatin-methacryloyl, which all showed high swelling responses to specific biomolecular sequence motifs, such as polymerizing and terminating DNA hairpins. These hydrogels may be used as programmed and autonomous untethered smart devices with biomolecular signals.

### Amine–Epoxidation Reaction

Ethylene glycol diglycidyl ether (EGDE) contains two epoxide groups on the structure, which can precipitate click reaction with nucleophilic groups such as amino, hydroxyl, sulfhydryl groups. It is widely used in the crosslinking of polysaccharides, proteins, and other biomolecules (Lu et al., 2006; He et al., 2020). While guanine (G) in the DNA contains many amino groups, the preparation of DNA-based hydrogels using EGDE as diepoxide crosslinkers has also attracted great attention (Orakdogen et al., 2010; Costa et al., 2011; Karacan et al., 2013). For instance, Topuz et al. used this principle and prepared DNA-based hydrogels by direct mixing of EGDE and DNA under alkaline condition by using EGDE as the crosslinking agent, TEMED as the catalyst, and salmon dsDNA as the skeleton material. The results showed that the elastic modulus of the obtained DNA-based hydrogel increased with the increase of EGDE content (Topuz and Okay, 2008, 2009).

Another study also reported a DNA/multiwalled carbon nanotube (MWCNT) hybrid hydrogel by crosslinking amine with EGDE. The authors first added short-chain salmon sperm DNA to the MWCNT, which could increase the solubility of MWCNT by hydrophobic interaction and π–π stacking. After ultrasonic treatment, uniform dispersion of the MWCNT-DNA complex was obtained. Then, free DNA with high molecular weight and a small amount of EGDE were added into this MWCNT dispersion. Thus, the DNA/MWCNT hybrid hydrogel could be formed by crosslinking of EGDE with amino groups in free DNA and DNA of MWCNT-DNA complex under alkaline and high-temperature conditions. The method is simple and efficient, which can meet the needs of mass production of DNA-based hydrogels, and has a good application prospect in the field of tissue engineering (Zinchenko et al., 2015). Meanwhile, a similar method was also used to prepare a highly porous DNA/MWCNT hybrid hydrogel (Ma et al., 2021). In this study, oil-in-water Pickering emulsion was first continuously produced inside DNA/MWCNT/EGDE mixture solution under shaking. With an increase in temperature, gelation of the mixture occurred because of the crosslinking reaction of EGDE. Then, a porous DNA/MWCNT hybrid hydrogel will be obtained by evaporating the oil phase through freeze-drying. This porous DNA-based hybrid hydrogel could be used for scavenging trace amounts of carcinogen polycyclic aromatic hydrocarbons (PAH), which has great potential applications in environmental protection.

**FIGURE 4:**
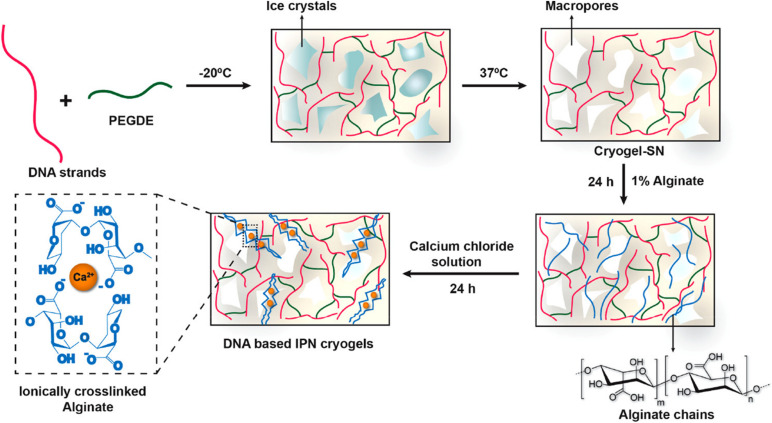
Sequential strategy for the development of DNA-based interpenetrating network cryogels from DNA and alginate. Reprinted with permission from Basu et al. (2020). Copyright (2020) American Chemical Society.

Besides EGDE, other substances with two epoxide groups, such as polyethylene glycol diglycidyl ether (PEGDE), could also be used as crosslinking agents. In a representative study, the authors developed a type of DNA-based hydrogel scaffold with an interpenetrating polymeric network (IPN) from DNA and alginate by amine–epoxidation reaction (Basu et al., 2020). The DNA strands were first covalently crosslinked by PEGDE and formed DNA-based cryogel at low temperatures (Figure 4). Then, alginate chains were absorbed into the macroporous cryogel network and formed interpenetrating network structures by ionic crosslinking with Ca^2+^, which significantly enhanced the toughness and energy dissipation of hydrogels. The resulting DNA-based IPN cryogels may be used as biomaterial scaffolds in tissue engineering applications.

### Other Interactions

In addition to those mentioned previously, other special interactions could also be used for the construction of DNA-based hydrogels. For instance, Lyu et al. (2018) constructed a novel stimuli-responsive DNA-based hydrogel controlled release system using hydrophobic interactions with liposomes as crosslinking agents. In this study, ssDNA-modified acrylate and acrylamide were first polymerized by free radical polymerization and then hybridized with complementary DNA sequence modified by cholesterol tetraethylene glycol to obtain polyacrylamide polymer with cholesterol side group. After mixing it with 1,2-dioleoyl-sn-glycerol-3-cholinophosphate (DOPC) liposome solution, the DNA-based hydrogel can be formed because of hydrophobic interaction between cholesterol and liposome bilayers. The gel–sol transformation characteristic of hydrogels also could be regulated by temperature, restriction endonuclease, and other factors. This hydrogel also has self-healing and injectable properties, showing good application prospects in the field of drug-controlled release.

Another study also designed a novel DNA-based composite hydrogel with small molecular ligand responsiveness using the specific binding of DNA binding protein to DNA (Christen et al., 2011). The selected protein is tetracycline inhibitor TetR, which can bind to the tetO-DNA motif. The protein and DNA chain were first covalently linked to the polyacrylamide linear polymer chain, respectively. After mixing, the composite hydrogel can be obtained, depending on the specific binding of TetR and tetO-DNA. In the presence of tetracycline, the hydrogel would gradually dissociate, thus controlling the release of internally embedded interleukin 4 model drug. This study is of great enlightening significance in the synthesis of biological hybrid materials with stimuli sensitivity such as drugs, metabolites, or toxins.

### Multiple Interactions

Moreover, by introducing multiple interactions or crosslinking into DNA-based hydrogels, the mechanical strength, responsiveness, and other properties of the formed hydrogels can be better regulated. In representative research, the authors designed a double crosslinked, injectable DNA-based hydrogel to improve the mechanical properties of the gel by taking advantage of hydrogen bonding and electrostatic interaction (Basu et al., 2018). DNA chains were first crosslinked by hydrogen bonding between complementary base pairs. And then, 2D silicate nanodisks with anisotropic charge distribution were introduced to generate additional network crosslinking points through electrostatic interaction with the DNA chains, thereby enhancing the mechanical elasticity of the DNA-based hydrogels. With the increase of the content of silicon nanodisks, the elasticity and yield stress of hydrogels would increase. On the other hand, they also used PEGDE as the crosslinking agent and designed a DNA-based nanocomposite hydrogel by amine–epoxidation reaction (Basu et al., 2019). To increase the durability and mechanical strength, silicate nanodisks were also introduced into the hydrogels together to form additional physical network points by electrostatic interaction. The resulting nanocomposite hydrogel could be used as a vehicle for the sustained delivery of a promising chemoattractant SDF-1α without compromising its biological activity.

A programmable mechanical responsive DNA-based hydrogel soft robotics was also developed based on the double-crosslinking mechanism (Zhao et al., 2019). A methacrylate-modified DNA linker sequence was first copolymerized with acrylamide and bis-acrylamide monomer through free radical double-bond polymerization reaction and formed a chemical crosslinked hydrogel. Then, pairs of DNA linkers within the hydrogel were connected by the adding bridge DNA strands via hydrogen bonding to form the double-crosslinked hydrogel. By regulating the length or sequence of DNA linkers, the shrinkage or responsiveness of this hydrogel could be controlled. Through combination with a bottom-up 3D printing technology, this DNA-based double-crosslinked hydrogel could be used as a responsive hydrogel palm with highly programmable movement.

**FIGURE 5:**
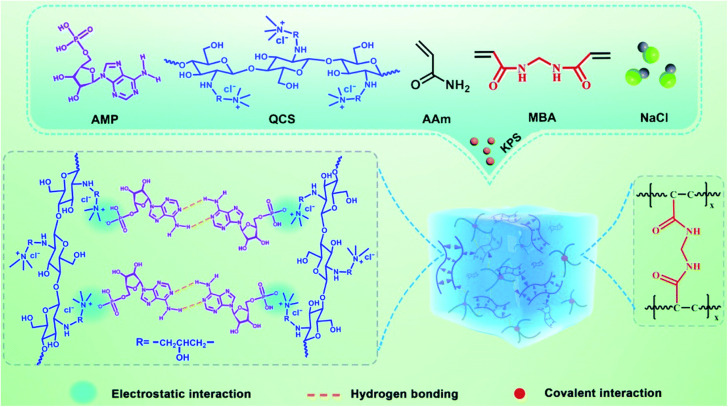
Schematic diagram of the preparation of DNA-inspired hydrogel mechanoreceptor. Reprinted with permission from Zhang et al. (2021). Copyright (2021) The Royal Society of Chemistry.

Moreover, Zhang et al. (2021) designed and developed a DNA-inspired hydrogel mechanoreceptor with skin-like perception and mechanical behavior through the triple combination of hydrogen bonding, electrostatic interaction, and covalent interaction. AMP was selected as a nucleotide model and mixed with quaternized chitosan (QCS), acrylamide, bis-acrylamide, and sodium chloride first. After double-bond polymerization, the hydrogel could be obtained (Figure 5). In this hydrogel, QCS was interacted with AMP via electrostatic interaction between phosphate groups and quaternary ammonium groups and forms a network structure by self-complementary hydrogen bonding between AMP. The interpenetrated network of polyacrylamide was constructed by covalent interaction and integrated with the QCS-AMP network through a large number of hydrogen bonds. The resulting skin-like hydrogels displayed high softness, fast self-recovery, good biocompatibility, and cycling stability.

## Biomedical Application of DNA-Based Hydrogels

Until now, there were a variety of DNA-based hydrogels with unique properties that had been constructed from different assembled DNA nanostructures or combined with novel functional materials or elements by a great diversity of crosslinking methods. The resulting pure DNA-based hydrogels or hybrid hydrogels have the advantages of excellent selection specificity, good structural designability, precise molecular recognition ability, and tunable responsiveness. Their physicochemical properties could also be facile tailored by the proper design of their building blocks. Thus, DNA-based hydrogels have aroused great concern in the biomedical area, especially in drug delivery, biosensing, and tissue engineering (Figure 1).

### Drug Delivery

Targeted delivery and controlled release of therapeutic molecules have been an important problem in the research of modern technology. They attracted extensive research on carriers, biocompatibility, biodegradability, and transport and release mode in different disease contexts. As an eminent skeleton material, DNA-based hydrogels have overcome a series of potential problems of targeted therapy systems because of their good biocompatibility, low toxicity, targeting, and other characteristics. It is believed that DNA-based hydrogels can be used as excellent drug carriers for the target treatment of many chronic diseases such as cancer *in vivo* (Lattuada et al., 2020; Mo et al., 2021).

In representative research, the authors believed that stimulation of innate immunity in the body was a long-lasting and effective treatment for cancer. Thus, they designed an immunostimulatory DNA-based injectable hydrogel harboring unmethylated cytosine-phosphate-guanine dinucleotides through the assembly of hexapod-like structured DNA. The cationic ovalbumin (ovalbumin OVG) could combine with hexapod-like structured DNA through electrostatic interaction. This pure DNA-based hydrogel could be used for rapid and accurate treatment in tumors to control the release of the vaccine ovalbumin antigen. The results showed that this method effectively inhibited the growth of tumor cells, which provided a great reference for cancer immunotherapy (Umeki et al., 2015).

To enhance the therapeutic effect on the tumor, a novel gold nanorod (AuNR)–based thermo–chemo combination therapy platform was developed utilizing DNA-based hydrogel (Song et al., 2015). They used a kind of X-shaped DNA with three sticky arms, which could form assembly hydrogel by hydrogen bonding. AuNRs, which are positively surface-charged, were stably incorporated into the hydrogel pores by electrostatic attractions. And doxorubicin, a DNA-binding anticancer drug with a strong binding affinity with DNA, was also loaded in this hydrogel. Because of the photo–thermal effects of AuNRs, this DNA-based hydrogel has unique melting characteristics by thermal denaturation under near-infrared, which could allow “on-demand”–type activation of the therapeutic combination action.

**FIGURE 6:**
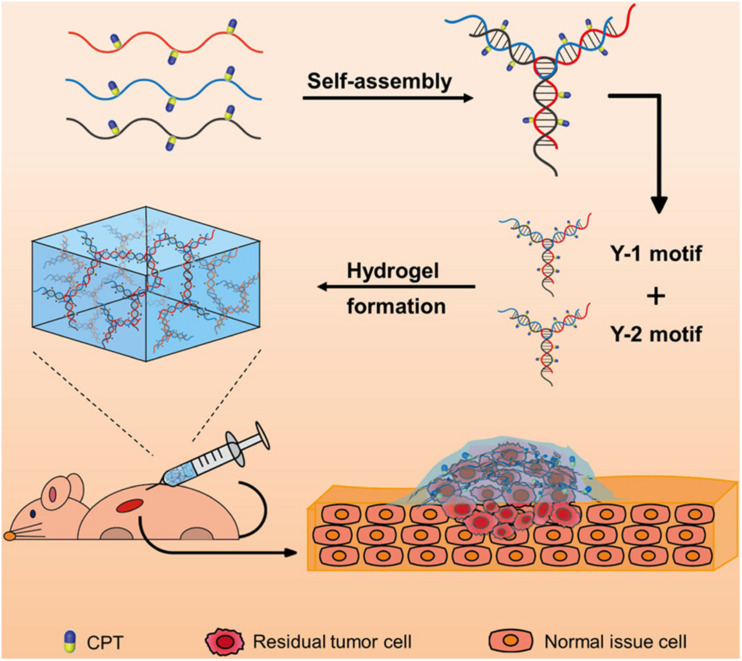
Schematic diagram of the injectable drug-conjugated DNA hydrogel for local chemotherapy to prevent tumor recurrence. Reprinted with permission from Zhang et al. (2020). Copyright (2020) American Chemical Society.

Clinically, patients will often face the risk of regrowth of tumor cells and tumor recurrence after surgical treatment, because of the possible residual cancer cells. To solve this problem, a DNA-based injectable hydrogel assembled by chemodrug-grafted DNA strands was designed for localized chemotherapy (Figure 6). A multitude of phosphorothioate oligonucleotide modification sites was first evenly introduced into two kinds of DNA strands, which could conjugate with carbonethyl bromide–modified model drug camptothecin (CPT), separately. The obtained two CPT-grafted DNAs will assemble into Y-shaped motifs separately and then assembled into hydrogels through hydrogen bonding. After injection, because of the presence of nucleases, this DNA-based hydrogel can gradually disassemble into nanoscale particles and be efficient uptake by cells. Together with the reduction responsive release of the conjugated CPT, this DNA-based hydrogel has high therapeutic efficacy against tumor recurrence while exhibiting low toxicity to normal tissues, which provides a promising approach for local adjuvant therapy in cancer treatment (Zhang et al., 2020).

### Biosensing

A biosensor is an important tool for medical examination and analysis. In the process of practice, people hope that the sensing tool has low cost and high detection sensitivity and is portable and easy to operate. As well known, many DNA-based hydrogels can respond rapidly to stimuli factors, such as pH, ions, and biomolecules in the external environment, so they are also widely used in the field of the biosensor. The sensing ability of DNA-based hydrogels can be achieved by changing the swelling volume, mechanical properties, crosslinking density, or the readout signal of released substances (Jonášová and Stokke, 2016; Khajouei et al., 2020).

**FIGURE 7:**
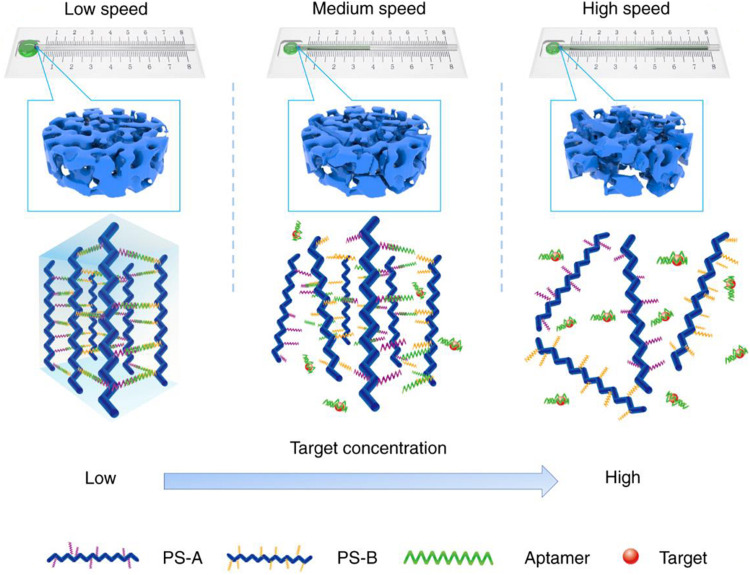
Schematic diagram of the working principle of the DNA-based hydrogel capillary self-driven regulator sensor. Reprinted under the terms and conditions of the Creative Commons CC BY License (Li F. et al., 2019). Copyright 2019, the authors, published by Springer Nature.

Representative research reported a facile and sensitive capillary self-driven regulator sensor, which can transform the analyte-induced small permeability change inside the DNA-based hydrogel into a visual signal (Li F. et al., 2019). For this purpose, the authors prepared a DNA-based hydrogel crosslinked by cocaine aptamer in a capillary tube subtly to block the tube. In the presence of cocaine, the cocaine aptamer can bind with cocaine, which will result in the degradation of hydrogel to a certain degree and increase the permeability of this hydrogel, thereby changing the flow velocity of the sample solution in the capillary tube (Figure 7). Therefore, the duration of the sample solution flowing through the capillary tube could be used to characterize the concentration of cocaine. This detection method of cocaine has a low detection limit (1.17 nM) and good selectivity, which opened a route for the design of visual quantitative sensors.

Another study also developed an enzyme-encapsulated DNA-based hydrogel innovative bioetching photoelectrochemical mode for highly sensitive and rapid detection of glucose (Zeng et al., 2020). The researchers prepared a GOx and HRP–doped DNA-based hydrogel by using the specific binding of biotin and avidin, in which HRP and GOx were *in situ* encapsulated. In the presence of glucose, GOx will oxidize glucose to form hydrogen peroxide, which is then further used as a substrate to participate in the bioetching reaction of cadmium sulfide (CdS) in the presence of HRP. The bioetching of CdS inhibits the electron transfer efficiency of In_2_O_3_ and CdS, which causes a change in photocurrent intensity. The results showed that the photocurrent intensity linear decreased with continuously increasing glucose concentration. This quantification method of glucose also has a satisfactory antijamming capability.

In the clinical detection of pathogenic microorganisms, polymerase chain reaction (PCR) and enzyme-linked immunosorbent assay technology are commonly used, which is fast, accurate, and specific. But it has certain limitations in the detection and diagnosis of latent pathogens. To improve the sensitivity and applicability of detection methods of pathogens, a DNA-based hydrogel microfluidic device formed by physical entanglement was prepared for rapid screening and detection of infectious pathogens (Na et al., 2018). The researchers first designed a kind of DNA template molecule with an asymmetric dumbbell shape and fixed it on the surface of the agarose beads, which were heavily filled inside the microfluidic device. When the target pathogen is encountered, the template DNA with pathogen-binding sites will hybridize with it, and the open template will be connected to form a closed dumbbell DNA template with the help of ligase. Moreover, under the action of Phi29 polymerase, RCA would occur. Over time, because of the narrow gap between adjacent beads, the dumbbell-shaped long-chain DNA will form hydrogel through physical entanglement, which will increase the surface area of microbeads, reduce the corresponding cross-sectional flow area, and prevent the flow of liquid between microbeads, resulting in a change in pressure. This method shortened the detection time of pathogens to less than 15 min, showing a good application prospect. They further prepared a similar DNA-based hydrogel for the rapid and accurate diagnosis of SARS-CoV-2 (severe acute respiratory syndrome coronavirus 2) (Kim et al., 2021).

### Tissue Engineering

Tissue engineering and cell culture *in vitro* are particularly important in tissue repair and regenerative medicine. In recent years, hydrogel has received wide attention in this field. Because of the excellent physical and chemical properties such as good biocompatibility, softness, and deformability, the hydrogel can provide a good matrix for cell culture and proliferation *in vitro* as a 3D skeleton material. Therefore, as a type of novel biomaterial, DNA-based hydrogel, which combines the biological characteristics of DNA with the skeleton function of hydrogel, has the advantage of designable responsiveness, biodegradability, and permeability and also has great potential in tissue engineering and cell culture.

Wang et al. (2017) developed a self-healing pure DNA-based supramolecular hydrogel to fabricate 3D tissue–like structures based on “brick-to-wall” technology. The DNA-based hydrogel bricks were formed with two monomers, Y-shaped DNA structures (Y) and linker (L) through corresponding sticky-ends’ matching. Cells such as HeLa cells and THP-1 cells can be encapsulated *in situ* and cultured in the hydrogel bricks. And then, two or more bricks could be easily piled together to form a designed 3D structure, and this process could be finished in seconds by self-healing. The cells inside can migrate freely and build more complicated multicellular structures. This method may benefit the large-scale production of artificial tissue in the future.

To explore the feasibility of DNA-based hydrogel as bioprinting scaffold material, the researchers designed two kinds of bioprinting inks, in which ink A contained peptide-DNA conjugate, which was synthesized by click reaction from azide ssDNA and polypeptide; ink B was used as the crosslinking agent, which was composed of dsDNA with two sticky ends. Through the *in situ* multilayer 3D bioprinting, two bioinks were alternately deposited, and the polypeptide-DNA–based hydrogel can be quickly crosslinked by hydrogen bonds in a few seconds. When AtT-20 cells were used as a model, they found that the printing process showed negligible damage to the cells, and the hydrogel had enough mechanical strength to provide physical support for the long-term cell cultures. The results show that it may be an ideal bioprinting material for the construction of designable 3D tissue–like constructs in tissue engineering (Li et al., 2015).

**FIGURE 8:**
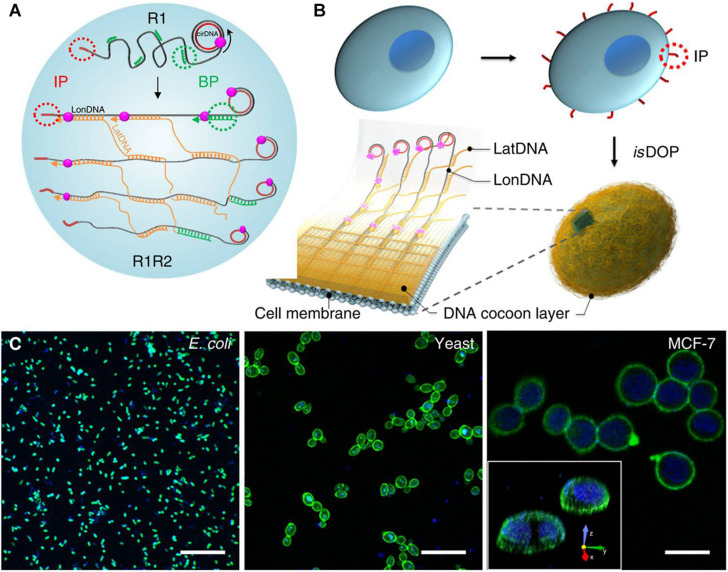
Schematic diagram of design and fabrication of flexible DNA-based hydrogel cocoons to encapsulate live cells: **(A,B)** the preparation of DNA-based hydrogel cocoons; **(C)** the scanning confocal microscope images of typical cell types encapsulated in the cocoons. Reprinted under the terms and conditions of the Creative Commons CC BY License (Gao et al., 2019). Copyright 2019, the authors, published by Springer Nature.

Encapsulation and manipulation of designated live cells freely have great potential application value in tissue engineering. Representative research designed an *in situ* DNA-oriented polymerization approach to weaving functional DNA-based hydrogel cocoons to encapsulate bacteria, yeast, or mammalian cells (Gao et al., 2019). An initiating primer was first attached to the cell membrane by covalent ligation or non-covalent insertion, which will initiate the rolling cycling replication and generates long and periodic DNA polymers (the longitude DNA) on the surface of cells. Then, the branched primer would initiate the branched replication that generates the second kind of ssDNA polymers (the latitude DNA). The formed longitude DNA and latitude DNA will automatically crosslink during the reaction through complementary pairing, and a DNA-based hydrogel cocoon would fabricate *in situ* on the cell surface (Figure 8). The results suggest that this encapsulation was biocompatible and highly efficient. This approach also allows us to precisely encode, handle, and sort the encapsulated cells.

### Other Applications

Because of the unique physicochemical properties, DNA-based hydrogels have been used in diverse biomedical applications. Besides the applications mentioned previously, DNA-based hydrogels can also be used for cell imaging. To improve the stability of hydrogels for better *in situ* cell immobilization and imaging, a type of transparent DNA-polyacrylamide double-network hydrogel with interpenetrating structures was designed through sequential fabrication (Cao et al., 2020). This DNA-based hydrogel was first prepared by hydrogen bonding of DNA linker and Y-scaffold, which could be used for *in situ* embedding and culture of cells. Before the imaging process, acrylamide and bis-acrylamide were introduced into the hydrogel and diffused through the DNA network. Then, an *in situ* double-bond polymerization takes place, and a covalently crosslinked second network was rapidly formed, which immobilized the cells and locked the DNA-based hydrogel network. Through this strategy, this hydrogel could be stable enough to withstand washing cycles and realize 3D visualization of cells in the culturing conditions *in situ*.

Cell-free protein synthesis has attracted wide attention in protein engineering. However, high cost and low yield have been the critical limiting problems in common cell-free protein production systems. To solve the problem, a low-cost DNA-based hybrid hydrogel was simply prepared from polyethylene glycol diacrylate (PEGDA) and DNA for protein synthesis (Cui et al., 2020). First, acrydite modified linear DNA chains that included all the necessary transcription elements for protein expression *in vivo* were prepared by PCR. Then, the acrydite modified DNA was mixed with PEGDA, and double-bond polymerization was occurred, which formed DNA-based hybrid hydrogels. The results showed that this DNA-based hybrid hydrogel system can enhance protein expression and serve as a repeated protein synthesis for tens of time, which provides a possibility for commercial protein production.

## Conclusion and Perspectives

In recent years, DNA-based materials have attracted widespread attention because of their excellent selective recognition ability and structural designability. On account of the combination of physicochemical properties of biomedical hydrogels and biological functions of DNA, DNA-based hydrogels have an excellent application prospect in the biomedical field, for instance, drug delivery, biosensing, tissue engineering, and so forth. Based on hydrogen bonding, physical entanglement, and enzymatic reaction, researchers constructed a variety of pure DNA-based hydrogels with excellent properties. Among them, because of the structural specificity of DNA, hydrogen bonding based on base complementary pairing is the main crosslinking method in pure DNA-based hydrogels. Through precise structural design and control, this kind of hydrogels usually has stable and controllable structure and shows dynamic stimulus-responsive behavior. Physical entanglement of long-chain DNA is another physical crosslinking method for the formation of pure DNA-based hydrogels. Because the long-chain DNA is usually prepared by RCA by the use of fragmented ssDNA as the primer, this method is simple and low-cost. While enzymatic reaction is a chemical crosslinking method for the construction of pure DNA-based hydrogel, because of the specificity of the enzymatic reaction, the resulting DNA-based hydrogels generally have higher mechanical strength and environmental stability. But it may need a relatively long crosslinking time.

Furthermore, by combining with other novel functional materials or elements, a large number of DNA-based hybrid hydrogels with improved physicochemical properties have been published through physical or chemical crosslinking, which greatly expanded the research field and application prospect of DNA-based hydrogels. The physical crosslinking actions of DNA-based hybrid hydrogels mainly consist of electrostatic interaction, coordination interaction, and π–π stacking effect, which are generally relatively simple and fast. The hybrid hydrogels formed by electrostatic interaction are usually obtained by the facile mixing of electropositive substances or particles with electronegative DNA, which does not need the accurate structural design of DNA. The formation of DNA-based hybrid hydrogels by the coordination interaction of metal ions is also a common method, and the resulting hydrogels usually have favorable ionic responsiveness, which is suitable for biosensing and drug-controlled release. Besides, the weak π–π stacking effect of aromatic substances with DNA could also be used to construct hybrid hydrogel, which expands the diversity of DNA-based hydrogel. On the other hand, the chemical crosslinking DNA-based hybrid hydrogels usually have relatively stable structure and mechanical strength. This crosslinking reaction mainly consists of double-bond polymerization reaction and amine–epoxidation reaction. In the hybrid hydrogels formed by double-bond polymerization, the DNA blocks are generally located in the branched chains and are not directly involved in the crosslinking reaction. And these DNA blocks are usually responsive to ions, compounds, or other DNA sequences, which would cause the change of swelling degree, crosslinking density, or other properties of the hydrogel. So this kind of hybrid hydrogels is widely used in biosensing. In contrast, in the hybrid hydrogels formed by amine–epoxidation reaction, the crosslinking reaction occurs directly on the DNA blocks, which endow these hydrogels with excellent mechanical strength and stability. Moreover, multiple interactions or crosslinking could also be introduced into DNA-based hydrogel, which gives it better adjustable properties.

However, it is worth noting that there are still many disadvantages or problems that need to be further studied and explored in the development of DNA-based hydrogels: (1) Because of the high cost of DNA, DNA-based hydrogels are still expensive to be prepared and are difficult to be produced in large quantities, making them hard to meet the needs of practical applications. Developing more facile synthetic methods and preparation of hybrid hydrogel are possible improvement strategies for cost reduction in the future. (2) Compared with other kinds of hydrogels, some DNA-based bulk hydrogels respond slowly and need to be further modified to achieve rapid response. Constructing DNA-based microgels, hydrogel microbeads, or membranes is a promising way to achieve rapid response. (3) DNA-based hydrogels, especially some pure hydrogels formed through physical crosslinking, have weak mechanical strength. Thus, it needs to be emphatically considered in the preparation process. Among them, the introduction of multiple interactions into the hydrogels is a feasible method. (4) Many DNA-based hydrogels, especially hybrid hydrogels, still involve some toxic substances, which limits their applications in the biomedical field. To solve this problem, we should develop more effective crosslinking methods and search for new safe and multifunctional hybrid materials. (5) The development of DNA-based hydrogel is still in the basic stage, which means it is not deep enough and needs to be further strengthened. Believe that with the development of related disciplines and the joint efforts of researchers in various fields, the performance of hydrogels will be continuously optimized and expanded through the reasonable design and construction of various multifunctional DNA-based hydrogels. The problems mentioned above will certainly be effectively solved, and the prospect of DNA-based hydrogels will also be broader.

## Author Contributions

XJ wrote the manuscript. XJ, XF, YuL, FL, JT, and YY revised the manuscript. YaL designed the work of review and revised the manuscript. All authors contributed to the article and approved the submitted version.

## Conflict of Interest

The authors declare that the research was conducted in the absence of any commercial or financial relationships that could be construed as a potential conflict of interest.

## References

[B1] Ahmed E. M. (2013). Hydrogel: preparation, characterization, and applications: a review. *J. Adv. Res.* 6 105–121. 10.1016/j.jare.2013.07.006 25750745 PMC4348459

[B2] Bae S. J. Song H. Jung G. Y. Cho S. W. Bae J. W. Um S. H. (2013). A superhydrophobic layer formed by fluoro-derivative-treated gold sheets on grown-up zinc oxide nanoparticles for a spherical DNA hydrogel. *Coll. Surf. B Biointerfaces* 111 342–345. 10.1016/j.colsurfb.2013.06.005 23838202

[B3] Basu S. Alkiswani A. R. Pacelli S. Paul A. (2019). Nucleic acid-based dual cross-linked hydrogels for in situ tissue repair via directional stem cell migration. *ACS Appl. Mater. Interfaces* 11 34621–34633. 10.1021/acsami.9b10074 31483598 PMC7291362

[B4] Basu S. Johl R. Pacelli S. Gehrke S. Paul A. (2020). Fabricating tough interpenetrating network cryogels with dna as the primary network for biomedical applications. *ACS Macro Lett.* 9 1230–1236. 10.1021/acsmacrolett.0c00448 35638638

[B5] Basu S. Pacelli S. Feng Y. Lu Q. H. Wang J. X. Paul A. (2018). Harnessing the noncovalent interactions of DNA backbone with 2D silicate nanodisks to fabricate injectable therapeutic hydrogels. *ACS Nano* 12 9866–9880. 10.1021/acsnano.8b02434 30189128 PMC6563937

[B6] Cao T. Y. Jia H. Y. Dong Y. C. Gui S. B. Liu D. S. (2020). In situ formation of covalent second network in a DNA supramolecular hydrogel and its application for 3D cell imaging. *ACS Appl. Mater. Interfaces* 12 4185–4192. 10.1021/acsami.9b11534 31896250

[B7] Chai Q. Y. Jiao Y. Yu X. J. (2017). Hydrogels for biomedical applications: their characteristics and the mechanisms behind them. *Gels* 3:6. 10.3390/gels3010006 30920503 PMC6318667

[B8] Chen J. Zhu Y. Liu H. J. Wang L. H. (2020). Tailoring DNA self-assembly to build hydrogels. *Top. Curr. Chem.* 378:32. 10.1007/s41061-020-0295-7 32146604

[B9] Christen E. H. Karlsson M. Kämpf M. M. Schoenmakers R. Gübeli R. J. Wischhusen H. M. (2011). Conditional DNA-protein interactions confer stimulus-sensing properties to biohybrid materials. *Adv. Funct. Mater.* 21 2861–2867. 10.1002/adfm.201100731

[B10] Clever G. H. Shionoya M. (2010). Metal–base pairing in DNA. *Coord. Chem. Rev.* 254 2391–2402. 10.1016/j.ccr.2010.04.014

[B11] Costa D. Valente A. J. M. Miguel M. G. Queiroz J. (2011). Gel network photodisruption: a new strategy for the codelivery of plasmid DNA and drugs. *Langmuir* 27 13780–13789. 10.1021/la2026285 21936554

[B12] Cui J. H. Wu D. Sun Q. Yang X. Z. Wang D. D. Zhuang M. (2020). A PEGDA/DNA hybrid hydrogel for cell-free protein synthesis. *Front. Chem.* 8:28. 10.3389/fchem.2020.00028 32133338 PMC7039859

[B13] Daly A. C. Riley L. Segura T. Burdick J. A. (2020). Hydrogel microparticles for biomedical applications. *Nat. Rev. Mater.* 5 20–43. 10.1038/s41578-019-0148-6 34123409 PMC8191408

[B14] Gačanin J. Synatschke C. V. Weil T. (2020). Biomedical applications of DNA-based hydrogels. *Adv. Funct. Mater.* 30:1906253. 10.1002/adfm.201906253

[B15] Gao T. Chen T. S. Feng C. He X. Mu C. L. Anzai J. I. (2019). Design and fabrication of flexible DNA polymer cocoons to encapsulate live cells. *Nat. Commun.* 10:2946. 10.1038/s41467-019-10845-2 31270421 PMC6610073

[B16] Geng J. H. Yao C. Kou X. H. Tang J. P. Luo D. Yang D. Y. (2018). A fluorescent biofunctional DNA hydrogel prepared by enzymatic polymerization. *Adv. Healthc. Mater.* 7:1700998. 10.1002/adhm.201700998 29280301

[B17] Geng L. J. Yu X. D. Li Y. J. Wang Y. Q. Wu Y. Q. Ren J. J. (2019). Instant hydrogel formation of terpyridine-based complexes triggered by DNA via non-covalent interaction. *Nanoscale* 11 4044–4052. 10.1039/c8nr08532c 30768104

[B18] Guo W. W. Qi X. J. Orbach R. Lu C. H. Freage L. Mironi-Harpaz I. (2014). Reversible Ag(+)-crosslinked DNA hydrogels. *Chem. Commun.* 50 4065–4068. 10.1039/c3cc49140d 24616906

[B19] He Q. F. Kusumi R. Kimura S. Kim U. J. Deguchi K. Ohki S. (2020). Highly swellable hydrogel of regioselectively aminated (1→3)-α-d-glucan crosslinked with ethylene glycol diglycidyl ether. *Carbohydr. Polym.* 237:116189. 10.1016/j.carbpol.2020.116189 32241412

[B20] Helwa Y. Dave N. Froidevaux R. Samadi A. Liu J. W. (2012). Aptamer-functionalized hydrogel microparticles for fast visual detection of mercury(II) and adenosine. *ACS Appl. Mater. Interfaces* 4 2228–2233. 10.1021/am300241j 22468717

[B21] Hu Y. Y. Xie D. Wu Y. Lin N. G. Song A. X. Hao J. C. (2017). Hydrogels based on Ag+-modulated assembly of 5’-adenosine monophosphate for enriching biomolecules. *Chemistry* 23 15721–15728. 10.1002/chem.201703180 28833801

[B22] Hu Y. Niemeyer C. M. (2019). DNA nanotechnology: from DNA nanotechnology to material systems engineering. *Adv. Mater.* 31:1970190. 10.1002/adma.201970190 30767279

[B23] Huang Y. S. Xu W. L. Liu G. Y. Tian L. L. (2017). A pure DNA hydrogel with stable catalytic ability produced by one-step rolling circle amplification. *Chem. Commun.* 53 3038–3041. 10.1039/c7cc00636e 28239729

[B24] Jonášová E. P. Stokke B. T. (2016). Bioresponsive DNA-co-polymer hydrogels for fabrication of sensors. *Curr. Opin. Coll. Interface Sci.* 26 1–8. 10.1016/j.cocis.2016.07.001

[B25] Jorge A. F. Eritja R. (2018). Overview of DNA self-assembling: progresses in biomedical applications. *Pharmaceutics* 10:268. 10.3390/pharmaceutics10040268 30544945 PMC6320858

[B26] Kahn J. S. Hu Y. W. Willner I. (2017). Stimuli-responsive DNA-based hydrogels: from basic principles to applications. *Acc. Chem. Res.* 50 680–690. 10.1021/acs.accounts.6b00542 28248486

[B27] Karacan P. Cakmak H. Okay O. (2013). Swelling behavior of physical and chemical DNA hydrogels. *J. Appl. Polym. Sci.* 128 3330–3337. 10.1002/app.38550

[B28] Khajouei S. Ravan H. Ebrahimi A. (2020). DNA hydrogel-empowered biosensing. *Adv. Coll. Interface Sci.* 275:102060. 10.1016/j.cis.2019.102060 31739981 PMC7094116

[B29] Khimji I. Kelly E. Y. Helwa Y. Hoang M. Liu J. W. (2013). Visual optical biosensors based on DNA-functionalized polyacrylamide hydrogels. *Methods* 64 292–298. 10.1016/j.ymeth.2013.08.021 23978515

[B30] Kim H. S. Abbas N. Shin S. (2021). A rapid diagnosis of SARS-CoV-2 using DNA hydrogel formation on microfluidic pores. *Biosens. Bioelectron.* 177:113005. 10.1016/j.bios.2021.113005 33486135 PMC7813512

[B31] Kurapati R. Reddy U. V. Raichur A. M. Suryaprakash N. (2016). Facile synthesis of graphene oxide/double-stranded DNA composite liquid crystals and hydrogels. *J. Chem. Sci.* 128 325–330. 10.1007/s12039-016-1043-y

[B32] Langford G. J. Raeburn J. Ferrier D. C. Hands P. J. W. Shaver M. P. (2019). Morpholino oligonucleotide cross-linked hydrogels as portable optical oligonucleotide biosensors. *ACS Sensors* 4 185–191. 10.1021/acssensors.8b01208 30592402

[B33] Lattuada E. Leo M. Caprara D. Salvatori L. Stoppacciaro A. Sciortino F. (2020). DNA-gel, novel nanomaterial for biomedical applications and delivery of bioactive molecules. *Front. Pharmacol.* 11:01345. 10.3389/fphar.2020.01345 33013376 PMC7500453

[B34] Lee J. B. Peng S. M. Yang D. Y. Roh Y. H. Funabashi H. Park N. (2012). A mechanical metamaterial made from a DNA hydrogel. *Nat. Nanotechnol.* 7 816–820. 10.1038/nnano.2012.211 23202472

[B35] Li C. Faulkner-Jones A. Dun A. R. Jin J. Chen P. Xing Y. Z. (2015). Rapid formation of a supramolecular polypeptide-DNA hydrogel for in situ three-dimensional multilayer bioprinting. *Angew. Chem.* 54 3957–3961. 10.1002/anie.201411383 25656851

[B36] Li F. Y. Lyu D. Y. Liu S. Guo W. W. (2020). DNA hydrogels and microgels for biosensing and biomedical applications. *Adv. Mater.* 32:e1806538. 10.1002/adma.201806538 31379017

[B37] Li F. Tang J. P. Geng J. H. Luo D. Yang D. Y. (2019). Polymeric DNA hydrogel: design, synthesis and applications. *Progress Polym. Sci.* 98:101163. 10.1016/j.progpolymsci.2019.101163

[B38] Li J. Mo L. T. Lu C. H. Fu T. Yang H. H. Tan W. H. (2016). Functional nucleic acid-based hydrogels for bioanalytical and biomedical applications. *Chem. Soc. Rev.* 45 1410–1431. 10.1039/c5cs00586h 26758955 PMC4775362

[B39] Li Y. Ma Y. Jiao X. Li T. Lv Z. Yang C. J. (2019). Control of capillary behavior through target-responsive hydrogel permeability alteration for sensitive visual quantitative detection. *Nat. Commun.* 10:1036. 10.1038/s41467-019-08952-1 30850603 PMC6408548

[B40] Lu C. H. Guo W. W. Qi X. J. Neubauer A. Paltiel Y. Willner I. (2015). Hemin-G-quadruplex-crosslinked poly-N-isopropylacrylamide hydrogel: a catalytic matrix for the deposition of conductive polyaniline. *Chem. Sci.* 6 6659–6664. 10.1039/c5sc02203g 29435215 PMC5802269

[B41] Lu S. S. Wang S. Zhao J. H. Sun J. Yang X. R. (2018). A pH-controlled bidirectionally pure DNA hydrogel: reversible self-assembly and fluorescence monitoring. *Chem. Commun.* 54 4621–4624. 10.1039/c8cc01603h 29671425

[B42] Lu X. L. Xu Y. H. Zheng C. Y. Zhang G. F. Su Z. (2006). Ethylene glycol diglycidyl ether as a protein cross-linker: a case study for cross-linking of hemoglobin. *J. Chem. Technol. Biotechnol.* 81 767–775. 10.1002/jctb.1441

[B43] Lyu D. Y. Chen S. S. Guo W. W. (2018). Liposome crosslinked polyacrylamide/DNA hydrogel: a smart controlled-release system for small molecular payloads. *Small* 14:1704039. 10.1002/smll.201704039 29479856

[B44] Ma G. Zhang K. N. Wang H. Q. Liang Z. D. Zhou L. Yan B. (2021). Versatile synthesis of a highly porous DNA/CNT hydrogel for the adsorption of carcinogen PAH. *Chem. Commun.* 57 2289–2292. 10.1039/d0cc07066a 33533382

[B45] Mao X. X. Mao D. S. Chen T. S. Jalalah M. Al-Assiri M. S. Harraz F. A. (2020). DNA hydrogel-based three-dimensional electron transporter and its application in electrochemical biosensing. *ACS Appl. Mater. Interfaces* 12 36851–36859. 10.1021/acsami.0c08064 32660232

[B46] Mo F. L. Jiang K. Zhao D. Wang Y. D. Song J. Tan W. H. (2021). DNA hydrogel-based gene editing and drug delivery systems. *Adv. Drug Deliv. Rev.* 168 79–98. 10.1016/j.addr.2020.07.018 32712197

[B47] Morán M. C. Miguel M. G. Lindman B. (2007). DNA gel particles: particle preparation and release characteristics. *Langmuir* 23 6478–6481. 10.1021/la700672e 17488045

[B48] Morya V. Walia S. Mandal B. B. Ghoroi C. Bhatia D. (2020). Functional DNA based hydrogels: development, properties and biological applications. *ACS Biomater. Sci. Eng.* 6 6021–6035. 10.1021/acsbiomaterials.0c01125 33449674

[B49] Na W. Nam D. Lee H. Shin S. (2018). Rapid molecular diagnosis of infectious viruses in microfluidics using DNA hydrogel formation. *Biosens. Bioelectron.* 108 9–13. 10.1016/j.bios.2018.02.040 29494886 PMC7125521

[B50] Nagahara S. Matsuda T. (1996). Hydrogel formation via hybridization of oligonucleotides derivatized in water-soluble vinyl polymers. *Polym. Gels Netw.* 4 111–127. 10.1016/0966-7822(96)00001-9

[B51] Orakdogen N. Erman B. Okay O. (2010). Evidence of strain hardening in DNA gels. *Macromolecules* 43 1530–1538. 10.1021/ma902558f

[B52] Park N. Um S. H. Funabashi H. Xu J. F. Luo D. (2009). A cell-free protein-producing gel. *Nat. Mater.* 8 432–437. 10.1038/nmat2419 19329993

[B53] Seeman N. C. Sleiman H. F. (2018). DNA nanotechnology. *Nat. Rev. Mater.* 3 1–23. 10.1038/natrevmats.2017.68

[B54] Shao Y. Jia H. Y. Cao T. Y. Liu D. S. (2017). Supramolecular hydrogels based on DNA self-assembly. *Acc. Chem. Res.* 50 659–668. 10.1021/acs.accounts.6b00524 28299927

[B55] Shi R. H. Fern J. S. Xu W. N. Jia S. S. Huang Q. Pahapale G. (2020). Multicomponent DNA polymerization motor gels. *Small* 16:e2002946. 10.1002/smll.202002946 32776420

[B56] Song J. Im K. Hwang S. Hur J. Nam J. Ahn G. O. (2015). DNA hydrogel delivery vehicle for light-triggered and synergistic cancer therapy. *Nanoscale* 7 9433–9437. 10.1039/c5nr00858a 25959856

[B57] Song J. Lee M. Kim T. Na J. Jung Y. Jung G. Y. (2018). A RNA producing DNA hydrogel as a platform for a high performance RNA interference system. *Nat. Commun.* 9:4331. 10.1038/s41467-018-06864-0 30337586 PMC6193956

[B58] Spicer C. D. (2020). Hydrogel scaffolds for tissue engineering: the importance of polymer choice. *Polym. Chem.* 11 184–219. 10.1039/c9py01021a

[B59] Topuz F. Okay O. (2008). Rheological behavior of responsive DNA hydrogels. *Macromolecules* 41 8847–8854. 10.1021/ma801414p

[B60] Topuz F. Okay O. (2009). Formation of hydrogels by simultaneous denaturation and cross-linking of DNA. *Biomacromolecules* 10 2652–2661. 10.1021/bm900585v 19658412

[B61] Um S. H. Lee J. B. Park N. Kwon S. Y. Umbach C. C. Luo D. (2006). Enzyme-catalysed assembly of DNA hydrogel. *Nat. Mater.* 5 797–801. 10.1038/nmat1741 16998469

[B62] Umeki Y. Mohri K. Kawasaki Y. Watanabe H. Takahashi R. Takahashi Y. (2015). Induction of potent antitumor immunity by sustained release of cationic antigen from a DNA-based hydrogel with adjuvant activity. *Adv. Funct. Mater.* 25 5758–5767. 10.1002/adfm.201502139

[B63] Wang Y. J. Shao Y. Ma X. Z. Zhou B. N. Faulkner-Jones A. Shu W. M. (2017). Constructing tissuelike complex structures using cell-laden DNA hydrogel bricks. *ACS Appl. Mater. Interfaces* 9 12311–12315. 10.1021/acsami.7b01604 28300395

[B64] Xing Y. Z. Cheng E. J. Yang Y. Chen P. Zhang T. Sun Y. W. (2011). Self-assembled DNA hydrogels with designable thermal and enzymatic responsiveness. *Adv. Mater.* 23 1117–1121. 10.1002/adma.201003343 21181766

[B65] Xu Y. X. Wu Q. Sun Y. Q. Bai H. Shi G. Q. (2010). Three-dimensional self-assembly of graphene oxide and DNA into multifunctional hydrogels. *ACS Nano* 4 7358–7362. 10.1021/nn1027104 21080682

[B66] Yang D. Y. Peng S. M. Hartman M. R. Gupton-Campolongo T. Rice E. J. Chang A. K. (2013). Enhanced transcription and translation in clay hydrogel and implications for early life evolution. *Sci. Rep.* 3:3165. 10.1038/srep03165 24196527 PMC3819617

[B67] Zeng R. J. Huang Z. L. Wang Y. K. Tang D. P. (2020). Enzyme-encapsulated DNA hydrogel for highly efficient electrochemical sensing glucose. *ChemElectroChem* 7 1537–1541. 10.1002/celc.202000105

[B68] Zhang J. Guo Y. Y. Pan G. F. Wang P. Li Y. H. Zhu X. Y. (2020). Injectable drug-conjugated DNA hydrogel for local chemotherapy to prevent tumor recurrence. *ACS Appl. Mater. Interfaces* 12 21441–21449. 10.1021/acsami.0c03360 32314901

[B69] Zhang K. K. Yam V. W. W. (2020). Platinum (II) non-covalent crosslinkers for supramolecular DNA hydrogels. *Chem. Sci.* 11 3241–3249. 10.1039/c9sc05910e 34122831 PMC8157491

[B70] Zhang Q. Liu X. Duan L. J. Gao G. H. (2021). A DNA-inspired hydrogel mechanoreceptor with skin-like mechanical behavior. *J. Mater. Chem. A* 9 1835–1844. 10.1039/d0ta11437e

[B71] Zhao Z. Wang C. Yan H. Liu Y. (2019). Soft robotics programmed with double crosslinking DNA Hydrogels. *Adv. Funct. Mater.* 29:1905911. 10.1002/adfm.201905911

[B72] Zhou L. P. Jiao X. Y. Liu S. Y. Hao M. D. Cheng S. Y. Zhang P. X. (2020). Functional DNA-based hydrogel intelligent materials for biomedical applications. *J. Mater. Chem. B* 8 1991–2009. 10.1039/c9tb02716e 32073097

[B73] Zinchenko A. Taki Y. Sergeyev V. G. Murata S. (2015). DNA-assisted solubilization of carbon nanotubes and construction of DNA-MWCNT cross-linked hybrid hydrogels. *Nanomaterials* 5 270–283. 10.3390/nano5010270 28347011 PMC5312845

